# Gallic Acid Potentiates Cisplatin Response in Cervical Cancer Cells Through Coordinated Cellular and Molecular Alterations

**DOI:** 10.3390/biology15110825

**Published:** 2026-05-23

**Authors:** Elif Ozan, Mehmet Cudi Tuncer, İlhan Özdemir

**Affiliations:** 1 Department of Gynecology and Obstetrics, Elif Ozan Practice, 06690 Ankara, Turkey; drelifozan@hotmail.com; 2 Department of Anatomy, Faculty of Medicine, Dicle University, 21280 Diyarbakır, Turkey; 3 Department of Histology and Embryology, Faculty of Medicine, Kahramanmaraş Sütçü İmam University, 46000 Kahramanmaraş, Turkey; ilhanozdemir32@hotmail.com

**Keywords:** gallic acid, cervical cancer, HeLa cells, apoptosis, caspase activity, synergism

## Abstract

Cisplatin (Cis) resistance and dose-limiting toxicity remain major challenges in the treatment of cervical cancer, necessitating the development of more effective combination strategies. The effects of gallic acid (GA), alone and in combination with Cis, were evaluated in HeLa cervical cancer cells using cell viability, apoptosis, gene expression, caspase activity, and cytokine profiling assays. Drug interactions were assessed using the Chou–Talalay method. The GA+Cis combination was associated with enhanced cytotoxicity compared to single-agent treatments, with combination index (CI) values ranging from 0.61 to 0.92, indicating synergistic interactions. Selectivity index (SI) values exceeding 2 at 48 h suggested preferential cytotoxicity toward cancer cells. The combination treatment significantly increased apoptotic cell populations and was accompanied by elevated caspase-3 and caspase-9 activities. Gene expression analysis revealed increased BAX and CASP3 levels, along with decreased BCL2 and BIRC5 expression, resulting in an increased BAX/BCL2 ratio. In addition, cell cycle analysis indicated accumulation in the G2/M phase, while cytokine profiling demonstrated reduced levels of pro-inflammatory cytokines and increased IL-10 levels under combination treatment conditions. These findings suggest that GA enhances the biological activity of Cis in cervical cancer cells through coordinated modulation of apoptosis, cell cycle dynamics, and inflammatory signaling. However, these results are based on in vitro observations, and further in vivo and mechanistic studies are required to confirm the therapeutic potential of this combination strategy.

## Simple Summary

Cervical cancer remains a major global health problem, and although cisplatin is widely used in treatment, its effectiveness is often limited by resistance and side effects. Natural compounds have gained attention as potential adjuvants to improve therapeutic outcomes. In this study, we investigated the effects of gallic acid, a plant-derived compound, in combination with cisplatin in cervical cancer cells. Our results showed that the combination more effectively reduced cancer cell viability and increased programmed cell death compared to either treatment alone. We also observed changes in genes and cellular pathways associated with cell survival and stress responses. These findings suggest that gallic acid may enhance the activity of cisplatin and could contribute to the development of more effective combination strategies. However, further studies are needed to confirm these effects in more complex biological systems.

## 1. Introduction

Cervical cancer remains one of the leading causes of cancer-related morbidity and mortality among women worldwide, ranking as the fourth-most commonly diagnosed malignancy, particularly in low- and middle-income countries [1,2]. Persistent infection with high-risk human papillomavirus (HPV), especially HPV-16 and HPV-18, is the primary etiological factor, promoting malignant transformation through functional inactivation of tumor suppressor proteins such as p53 and retinoblastoma (Rb) by the viral oncoproteins E6 and E7 [3,4]. Despite advances in surgical and radiotherapeutic approaches, platinum-based chemotherapy remains a cornerstone in the management of advanced cervical cancer. Among these agents, Cis (cis-diamminedichloroplatinum II; Cis) is widely used due to its ability to induce DNA cross-linking and apoptosis. However, its clinical application is limited by dose-dependent toxicities and the development of chemoresistance [5,6]. These limitations highlight the need for alternative or combinational strategies to improve therapeutic efficacy while reducing adverse effects [7].

Natural compounds, particularly plant-derived polyphenols, have attracted considerable attention as potential anticancer agents due to their relatively low toxicity and diverse biological activities [8,9]. Gallic acid (GA; 3,4,5-trihydroxybenzoic acid) is a naturally occurring phenolic compound present in various fruits, teas, and plant extracts. Previous studies have reported that GA exhibits antioxidant, anti-inflammatory, and anticancer properties across multiple cancer types [10,11]. Previous experimental studies have further suggested that GA may influence oxidative stress-related pathways, mitochondrial function, apoptosis, and cell cycle regulation in cancer cells [12,13]. However, these observations appear to be highly context-dependent and may vary according to the cellular model, treatment conditions, and experimental design. Therefore, the precise upstream molecular events underlying GA-mediated anticancer effects remain incompletely understood.

Apoptosis plays a critical role in cancer development and therapeutic response. Dysregulation of apoptotic signaling pathways contributes to tumor progression and resistance to chemotherapy [14]. The balance between pro-apoptotic proteins such as BAX and anti-apoptotic proteins such as BCL-2 is a key determinant of mitochondrial outer membrane permeabilization and subsequent caspase activation [15]. Caspase-9 and caspase-3 function as initiator and executioner caspases, respectively, in the intrinsic apoptotic pathway [16]. In addition, survivin (BIRC5), a member of the inhibitor of apoptosis protein (IAP) family, suppresses caspase activity and is frequently overexpressed in cancer cells, contributing to treatment resistance and poor prognosis [17].

Although the anticancer effects of GA have been demonstrated in various experimental models, its potential to enhance the efficacy of Cis, particularly in cervical cancer cells, has not been fully elucidated. In addition, the extent to which GA modulates apoptosis-related gene expression, caspase activity, inflammatory cytokine responses, and cell cycle progression in combination with Cis remains unclear. Given the increasing interest in combination-based therapeutic strategies, quantitative evaluation of drug interactions is essential. In this context, the Chou–Talalay method provides a widely used framework for distinguishing synergistic, additive, and antagonistic effects between combined agents [18].

Based on these considerations, we hypothesized that the combination of GA and Cis would enhance anticancer effects in HeLa cervical cancer cells by promoting apoptosis and modulating key molecular pathways associated with cell survival. Accordingly, the aim of this study was to investigate the effects of GA and Cis, administered alone and in combination, on cell viability, apoptosis, cell cycle distribution, apoptosis-related gene expression, caspase activity, and cytokine profiles, as well as to evaluate potential drug interactions using CI analysis.

## 2. Materials and Methods

### 2.1. Cell Line and Culture Conditions

The human cervical cancer cell line HeLa (ATCC^®^ CCL-2™) and the human immortalized keratinocyte cell line HaCaT (CLS Cell Line Service GmbH, Eppelheim, Germany) were used in this study. HeLa cells were cultured in Dulbecco’s Modified Eagle Medium (DMEM; Gibco, Thermo Fisher Scientific, MA, USA) supplemented with 10% fetal bovine serum (FBS; Gibco, Thermo Fisher Scientific, Waltham, MA, USA) and 1% penicillin–streptomycin (Sigma-Aldrich, St. Louis, MO, USA). HaCaT cells were maintained under identical culture conditions. All cells were incubated at 37 °C in a humidified atmosphere containing 5% CO_2_ and were used for experiments during the logarithmic growth phase. Cells were routinely monitored for morphology and confluency prior to treatment. The HaCaT cell line was included as a non-tumorigenic control model to assess the selectivity of GA and Cis toward cancer cells.

### 2.2. Drug Preparation and Administration

Gallic acid (GA; 3,4,5-trihydroxybenzoic acid, purity ≥ 99%; Sigma-Aldrich, USA) was dissolved in dimethyl sulfoxide (DMSO; Sigma-Aldrich) to prepare a 100 mM stock solution. Cisplatin (Cis; Sigma-Aldrich) was dissolved in sterile saline to obtain a 10 mM stock solution. All stock solutions were aliquoted and stored at −20 °C, protected from light. Working concentrations were freshly prepared by diluting stock solutions in culture medium immediately prior to use. The final concentration of DMSO in all treatment groups did not exceed 0.1%, and the same concentration was used in control groups to exclude solvent-related effects. HeLa cells were seeded into 96-well plates at a density of 5 × 10^3^ cells per well and allowed to adhere for 24 h prior to treatment. Cells were then exposed to increasing concentrations of GA (10–500 µM) and Cis (0.5–20 µM) for 24 and 48 h. The selected concentration ranges were based on preliminary experiments and were used to determine dose–response relationships and half-maximal inhibitory concentration (IC_50_) values. HaCaT cells were included primarily to evaluate the selective cytotoxicity profile of GA and Cis treatments relative to non-tumorigenic cells. Subsequent mechanistic analyses were performed exclusively in HeLa cells to focus on cervical cancer-specific molecular responses.

### 2.3. Cell Viability

Cell viability was evaluated using the MTT assay. HeLa and HaCaT cells were seeded into 96-well plates at a density of 5 × 10^3^ cells per well and allowed to attach for 24 h prior to treatment. Cells were then exposed to increasing concentrations of GA (10–500 µM) or Cis (0.5–20 µM) for 24 and 48 h. Following treatment, 10 µL of MTT solution (5 mg/mL) was added to each well and incubated for 4 h at 37 °C. The resulting formazan crystals were dissolved in 150 µL of DMSO, and absorbance was measured at 570 nm with a reference wavelength of 630 nm using a microplate reader. Cell viability was expressed as a percentage relative to the vehicle-treated control group. Dose–response curves were generated, and IC_50_ values were calculated using nonlinear regression analysis (GraphPad Prism 9.0). To evaluate selective cytotoxicity, the SI was calculated as the ratio of IC_50_ values in HaCaT cells to those in HeLa cells (SI = IC_50_ HaCaT/IC_50_ HeLa), where SI > 2 was considered indicative of preferential cytotoxicity toward cancer cells. All experiments were performed with three independent biological replicates. For subsequent mechanistic experiments, HeLa cells were treated with GA (100 µM), Cis (3.5 µM), or their combination (100 µM GA + 3.5 µM Cis) for 48 h. These concentrations were selected based on the CI/isobologram analyses, which demonstrated synergistic interactions at fixed-ratio combinations while using a lower effective Cis concentration than the calculated single-agent IC_50_ value.

### 2.4. Combination Index and Isobologram Analysis

The interaction between GA and Cis was evaluated using the Chou–Talalay method, which is based on the median-effect principle. Drug combinations were applied at fixed ratios determined according to their respective IC_50_ values. For combination experiments, GA and Cis were applied at fixed ratios based on their respective 48 h IC_50_ values in HeLa cells. The tested combinations included GA concentrations of 40, 60, 80, 100, 120, 140, and 160 µM combined with Cis concentrations of 1.8, 2.4, 3.0, 3.5, 3.9, 4.2, and 4.5 µM, respectively. These concentration pairs were selected to generate a range of fraction affected (Fa) values for CI analysis according to the Chou–Talalay method. Based on the CI analysis, the combination consisting of 100 µM GA and 3.5 µM Cis was selected for subsequent apoptosis, cell cycle, immunocytochemistry, RT-qPCR, caspase activity, and cytokine analyses. Cell viability data obtained from the MTT assay were used to calculate Fa for each treatment condition. CI values were then computed using CompuSyn software (ComboSyn Inc., Paramus, NJ, USA, version 1.0) and independently verified with Combenefit software (Cambridge Cancer Centre, Cambridge, UK, version 2.021). CI values were interpreted as follows: CI < 1 indicates synergism, CI = 1 indicates an additive effect, and CI > 1 indicates antagonism. CI values were calculated across a range of effect levels to assess the consistency of drug interactions. Isobologram analyses were generated to visually represent the interaction between GA and Cis at selected effect levels.

### 2.5. Apoptosis and Cell Cycle Analysis

Apoptosis was evaluated using an Annexin V-FITC/propidium iodide (PI) staining kit (BD Biosciences, San Jose, CA, USA) according to the manufacturer’s instructions. Briefly, treated cells were harvested, washed twice with cold phosphate-buffered saline (PBS), and resuspended in 1× binding buffer at a density of 1 × 10^5^ cells/mL. Cells were incubated with Annexin V-FITC and PI (5 µL each) for 15 min at room temperature in the dark. Samples were analyzed within 1 h using a BD FACSCanto II flow cytometer (BD Biosciences, San Jose, CA, USA). At least 10,000 events were acquired per sample. Data were processed using FlowJo software (version X, BD Biosciences), and cell populations were classified as viable (Annexin V^−^/PI^−^), early apoptotic (Annexin V^+^/PI^−^), late apoptotic (Annexin V^+^/PI^+^), and necrotic (Annexin V^−^/PI^+^). For cell cycle analysis, cells were fixed in 70% ethanol at −20 °C for 12 h, washed with PBS, and incubated with propidium iodide (50 µg/mL) and RNase A (100 µg/mL) for 30 min at room temperature. DNA content was analyzed using a flow cytometer, and cell cycle distribution (G0/G1, S, and G2/M phases) was determined using ModFit LT software (Verity Software House, Topsham, ME, USA, version 5.0). The sub-G1 population was also evaluated as an indicator of apoptotic DNA fragmentation. All experiments were performed in three independent biological replicates.

### 2.6. NucBlue Immunocytochemistry Staining

Nuclear morphological changes associated with apoptosis were evaluated using NucBlue™ Live ReadyProbes™ Reagent (Hoechst 33342; Thermo Fisher Scientific, USA). Following treatment, cells were washed with phosphate-buffered saline (PBS) and fixed with 4% paraformaldehyde for 15 min at room temperature. Cells were then permeabilized using 0.1% Triton X-100 for 10 min and washed with PBS. Subsequently, NucBlue reagent was added according to the manufacturer’s instructions and incubated for 20 min in the dark. After washing three times with PBS, samples were mounted and examined under a fluorescence microscope (Zeiss Axio Scope, Carl Zeiss Microscopy GmbH, Jena, Germany) using a DAPI filter. Images were captured from at least five randomly selected fields per sample, and nuclear morphology was analyzed using ImageJ software (National Institutes of Health (NIH), Bethesda, MD, USA, version 1.54). Cells exhibiting characteristic apoptotic features, including chromatin condensation, nuclear fragmentation, and membrane blebbing, were identified and semi-quantitatively evaluated.

### 2.7. β-Tubulin Immunocytochemistry Staining

HeLa cells were seeded onto sterile coverslips at a density of 1 × 10^5^ cells per well in 24-well plates and allowed to adhere for 24 h. Cells were then treated with GA, Cis, or their combination (GA+Cis) for 48 h. Following treatment, cells were fixed with 4% paraformaldehyde for 15 min at room temperature, permeabilized with 0.1% Triton X-100 for 10 min, and blocked with 1% bovine serum albumin (BSA) for 30 min. Cells were incubated overnight at 4 °C with a primary antibody against β-tubulin (1:400; Cell Signaling Technology, Danvers, MA, USA; Cat# 2146), followed by incubation with an Alexa Fluor 488-conjugated secondary antibody (1:1000; [manufacturer]) for 1 h at room temperature in the dark. Nuclei were counterstained with Hoechst 33342 (1 µg/mL) for 5 min. Samples were mounted and visualized using a fluorescence microscope (Zeiss Axio Scope, Germany) at 400× magnification. Images were acquired from multiple randomly selected fields, and β-tubulin filament organization was qualitatively and semi-quantitatively assessed using ImageJ software. Changes in microtubule architecture, including filament disorganization, fragmentation, and reduced network integrity, were evaluated relative to the control group. All experiments were performed in three independent biological replicates.

### 2.8. RNA Isolation and Quantitative RT-PCR (RT-qPCR)

Total RNA was extracted using TRIzol™ Reagent (Thermo Fisher Scientific, USA) according to the manufacturer’s instructions. RNA concentration and purity were determined using a NanoDrop 2000 spectrophotometer (Thermo Fisher Scientific), and only samples with an A260/A280 ratio between 1.8 and 2.0 were used for further analysis. Complementary DNA (cDNA) was synthesized from 1 µg of total RNA using the High-Capacity cDNA Reverse Transcription Kit (Applied Biosystems, Foster, CA, USA), following the manufacturer’s protocol. Quantitative PCR (qPCR) was performed using SYBR Green Master Mix on a StepOnePlus™ Real-Time PCR System (Applied Biosystems). The amplification conditions consisted of an initial denaturation step at 95 °C for 10 min, followed by 40 cycles of denaturation at 95 °C for 15 s and annealing/extension at 60 °C for 1 min. A melt curve analysis was performed at the end of each run to confirm the specificity of amplification. The expression levels of BAX, BCL2, CASP3, and BIRC5 (survivin) were quantified, with GAPDH used as the internal reference gene. Relative gene expression levels were calculated using the 2^−ΔΔCt^ method. All reactions were performed in triplicate, and experiments were conducted using three independent biological replicates.

### 2.9. Caspase-3 and Caspase-9 Enzyme Activity

Caspase-3 and caspase-9 activities were determined using commercially available colorimetric assay kits (Caspase-3 Activity Kit, Catalog No. CASP3C; Caspase-9 Activity Kit, Catalog No. APT173; Sigma-Aldrich, USA), following the manufacturer’s instructions. Briefly, treated cells were harvested and lysed in the provided lysis buffer. Protein concentrations were quantified using the bicinchoninic acid (BCA) assay (Thermo Fisher Scientific), and equal amounts of protein (50 µg) were used for each reaction. Samples were incubated with specific chromogenic substrates, Ac-DEVD-pNA for caspase-3 and LEHD-pNA for caspase-9, at 37 °C for the recommended incubation period. The release of p-nitroaniline (pNA) was measured at 405 nm using a microplate reader. Enzyme activity was calculated and expressed as nmol/min/mg protein. All experiments were performed in three independent biological replicates.

### 2.10. Cytokine Level Determination (ELISA)

The levels of IL-6, IL-8, TNF-α, and IL-10 in cell culture supernatants were quantified using commercially available ELISA kits (R&D Systems, Minneapolis, MN, USA) according to the manufacturer’s instructions. Following treatment, culture supernatants were collected, centrifuged at 300× *g* for 10 min to remove cellular debris, and stored at −80 °C until analysis. ELISA assays were performed using standard protocols, and absorbance was measured at 450 nm using a microplate reader. Cytokine concentrations were calculated from standard curves generated for each analyte and expressed as pg/mL. All measurements were performed in triplicate across three independent biological replicates.

### 2.11. Predicting Potential Targets of Gallic Acid

Potential molecular targets of GA were predicted using the SwissTargetPrediction database (http://www.swisstargetprediction.ch; accessed on 10 April 2026). Genes associated with cervical cancer were retrieved from the GeneCards (https://www.genecards.org; accessed on 10 April 2026) and OMIM (https://omim.org; accessed on 10 April 2026) databases. Duplicate entries were removed, and a comprehensive list of disease-related genes was compiled. To improve prediction reliability, only targets with a defined probability threshold were considered. The intersection between predicted GA targets and cervical cancer-associated genes was identified using Venn diagram analysis. The overlapping gene set was used for subsequent bioinformatic analyses.

### 2.12. Protein–Protein Interaction (PPI) Network

The PPI network was constructed using the STRING database (https://string-db.org; version 12.0; accessed on 10 April 2026) based on the overlapping gene set identified from the intersection of predicted GA targets and cervical cancer-associated genes. The analysis was performed with the organism set to *Homo sapiens*, and an interaction confidence score ≥ 0.7 (high confidence) was applied as the threshold. Both known and predicted interactions were included in the network. The resulting interaction network was imported into Cytoscape software (version 3.10.1; https://cytoscape.org; accessed on 10 April 2026) for visualization and topological analysis. Key network parameters, including degree centrality, betweenness centrality, and closeness centrality, were calculated using the NetworkAnalyzer plugin. Genes with the highest degree values were identified as hub nodes within the network and were selected for further analysis.

### 2.13. Gene Ontology (GO) and KEGG Pathway Analysis

GO and Kyoto Encyclopedia of Genes and Genomes (KEGG) pathway enrichment analyses were performed using the clusterProfiler package (version 4.10.0) in R software (version 4.3.1). The analyses were conducted to explore the potential biological functions and signaling pathways associated with the overlapping gene set. GO enrichment analysis included three categories: biological process (BP), molecular function (MF), and cellular component (CC). KEGG pathway analysis was performed using the KEGG database (https://www.kegg.jp; accessed on 10 April 2026). A corrected *p*-value (Benjamini–Hochberg method) of < 0.05 was considered statistically significant. Enrichment results were visualized using bubble plots and network diagrams generated in R.

### 2.14. Statistical Analysis

Data from at least three independent experiments are expressed as mean ± SD. Differences between groups were assessed using one-way Analysis of Variance (ANOVA) with Tukey’s post hoc test, and comparisons between two groups were performed using an independent *t*-test. Statistical significance was set at *p* < 0.05. Analyses were performed using SPSS 26.0 and GraphPad Prism 9.0.

## 3. Results

### 3.1. Cytotoxic Effect and Selectivity Index of GA and Cis in HeLa and HaCaT Cells

**Figure 1:**
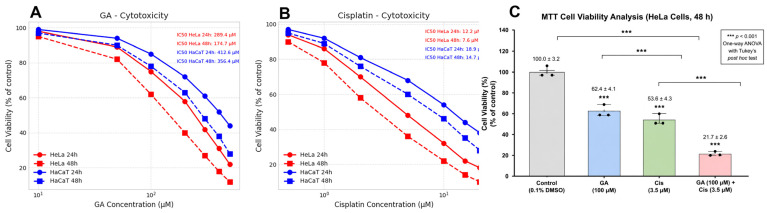
Dose–response curves and viability analysis of GA and Cis in HeLa and HaCaT cells following 24 and 48 h treatment. (**A**) Dose-dependent cytotoxic effects of GA in HeLa and HaCaT cells. (**B**) Dose-dependent cytotoxic effects of Cis in HeLa and HaCaT cells. IC_50_ values were calculated from MTT assay data and are indicated within each panel. (**C**) Representative MTT-based viability analysis of HeLa cells treated for 48 h with GA (100 µM), Cis (3.5 µM), or their combination. The GA+Cis combination significantly reduced cell viability compared with single-agent treatments, supporting the synergistic interaction identified by CI and isobologram analyses. Cell viability is expressed as a percentage relative to the vehicle-treated control. Data are presented as mean ± standard deviation (SD) from three independent biological replicates (*n* = 3). Statistical analysis was performed using one-way ANOVA followed by Tukey’s post hoc test (*** *p* < 0.001).

The cytotoxic effects of GA and Cis on HeLa and HaCaT cells were evaluated using the MTT assay. Both agents reduced cell viability in a dose- and time-dependent manner in both cell lines (Figure 1). However, the reduction in viability was more pronounced in HeLa cells compared to HaCaT cells. The IC_50_ values for GA at 24 h were 289.4 µM in HeLa cells and 412.6 µM in HaCaT cells, while the corresponding 48 h IC_50_ values were 174.7 µM and 356.4 µM, respectively. For Cis, the IC_50_ values were 12.2 µM (24 h) and 7.6 µM (48 h) in HeLa cells, compared to 18.9 µM and 14.7 µM in HaCaT cells. To assess selective cytotoxicity, the SI was calculated. The SI values for GA were 1.42 (24 h) and 2.55 (48 h), while those for Cis were 1.54 (24 h) and 2.53 (48 h). Notably, SI values exceeding 2 at 48 h suggest preferential cytotoxicity toward HeLa cancer cells relative to non-tumorigenic HaCaT cells. Based on the 48 h IC_50_ values and CI/isobologram analyses, GA (100 µM), Cis (3.5 µM), and their combination (100 µM GA + 3.5 µM Cis) were selected for subsequent mechanistic and molecular analyses.

### 3.2. Combination Index and Isobologram Analysis Findings

**Figure 2:**
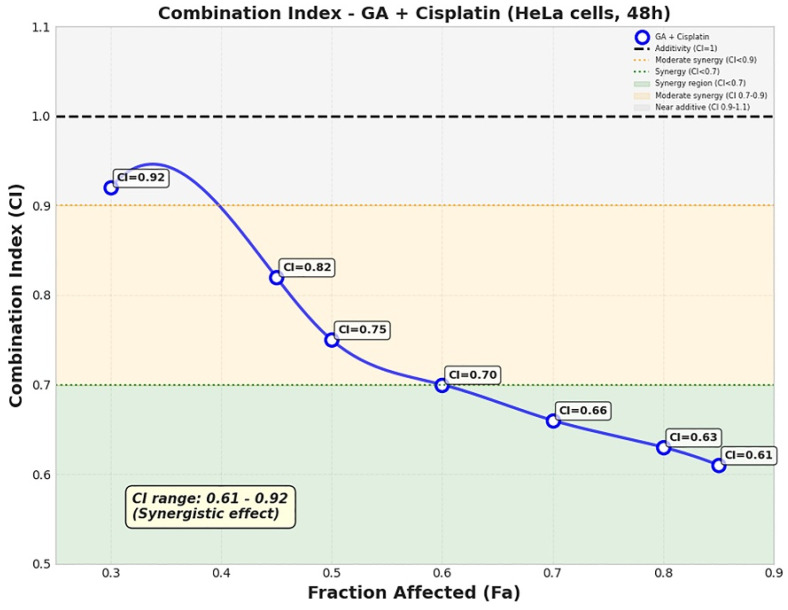
CI–Fa plot for the combination of GA and Cis in HeLa cells at 48 h. Fixed-ratio combinations were prepared based on IC_50_ values, and CI values were calculated using the Chou–Talalay method. CI values ranging from 0.61 to 0.92 indicate synergistic interactions across a range of effect levels (CI < 1, synergism; CI = 1, additive effect; CI > 1, antagonism). The tested fixed-ratio combinations were generated according to the respective 48 h IC_50_ values of GA and Cis.

**Figure 3:**
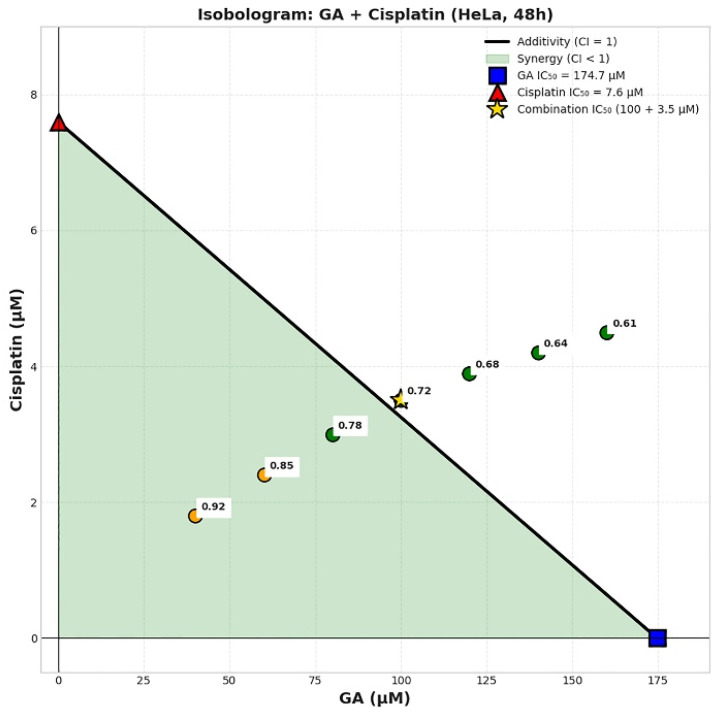
Isobologram analysis of the combination of GA and Cis in HeLa cells at 48 h. The solid diagonal line represents the theoretical line of additivity (CI = 1), while the shaded area indicates the synergistic interaction region (CI < 1). Combination data points generated from fixed-ratio treatments are shown together with their corresponding CI values. Overall, the combination treatments demonstrated predominantly synergistic interactions, consistent with the Chou–Talalay CI analysis. The representative combination selected for downstream mechanistic analyses consisted of 100 µM GA + 3.5 µM Cis.

The interaction between GA and Cis was evaluated using the Chou–Talalay method. In fixed-ratio combinations based on IC_50_ values, CI values ranged from 0.61 to 0.92 across different Fa levels (Figure 2). CI values below 1 indicate synergistic interactions according to the Chou–Talalay model. In the present study, CI values remained below 1 across a broad range of effect levels, supporting a predominantly synergistic interaction between GA and Cis in HeLa cells. The degree of interaction varied depending on Fa, with relatively stronger synergistic effects observed at higher Fa levels. Isobologram analysis further supported these findings, with most combination data points distributed within the synergistic interaction region below the theoretical line of additivity (Figure 3). Based on the CI and isobologram analyses, GA (100 µM), Cis (3.5 µM), and their combination (100 µM GA + 3.5 µM Cis) were selected for subsequent mechanistic and molecular analyses.

In the isobologram analysis, most combination data points were distributed within the synergistic interaction region below the line of additivity, supporting a synergistic interaction between GA and Cis at the tested concentrations (Figure 3). This observation is consistent with the CI analysis results obtained using the Chou–Talalay method. In addition, matrix-based analysis performed using Combenefit software suggested the presence of synergistic interactions across a range of dose combinations.

### 3.3. Flow Cytometry Analysis of Apoptosis and Cell Cycle

**Figure 4:**
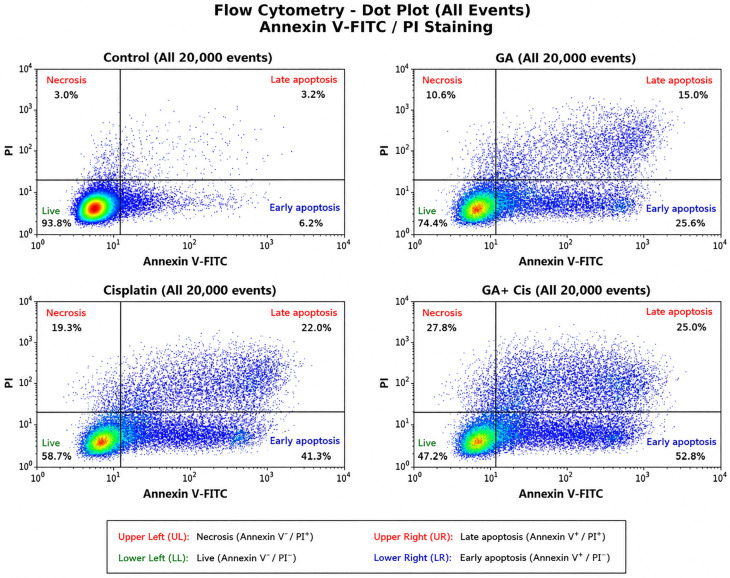
Flow cytometry analysis of apoptosis in HeLa cells following treatment with GA (100 µM), Cis (3.5 µM), and their combination (100 µM GA + 3.5 µM Cis) for 48 h. Representative Annexin V-FITC/PI dot plots are shown. Cells were classified as viable (Annexin V^−^/PI^−^), early apoptotic (Annexin V^+^/PI^−^), late apoptotic (Annexin V^+^/PI^+^), and necrotic (Annexin V^−^/PI^+^). The percentage of early apoptotic cells increased from 6.2 ± 0.8% in the control group to 25.6 ± 2.1% (GA), 41.3 ± 2.7% (Cis), and 52.8 ± 3.4% in the combination group. In parallel, the proportion of late apoptotic cells increased from 3.2% in the control group to 15.0% (GA), 22.0% (Cis), and 25.0% following combination treatment. These findings indicate that combined GA and Cis treatment enhanced apoptotic cell death more effectively than either single-agent treatment alone (*p* < 0.001).

Flow cytometric analysis using Annexin V-FITC/PI double staining demonstrated that treatment with GA (100 µM), Cis (3.5 µM), and their combination (100 µM GA + 3.5 µM Cis) increased apoptotic cell populations in HeLa cells (Figure 4). The proportion of early apoptotic cells (Annexin V^+^/PI^−^) increased from 6.2 ± 0.8% in the control group to 25.6 ± 2.1% following GA treatment and 41.3 ± 2.7% following Cis treatment, reaching 52.8 ± 3.4% in the GA+Cis combination group (*p* < 0.001). Similarly, late apoptotic cell populations (Annexin V^+^/PI^+^) were elevated in treated groups, increasing from 3.2 ± 0.5% in controls to 15.0 ± 1.4%, 22.0 ± 1.9%, and 25.0 ± 2.1% following GA, Cis, and combination treatment, respectively. Overall, the combination treatment produced the highest total apoptotic cell fraction (early + late apoptosis), indicating an enhanced apoptotic response compared with single-agent treatments.

**Figure 5:**
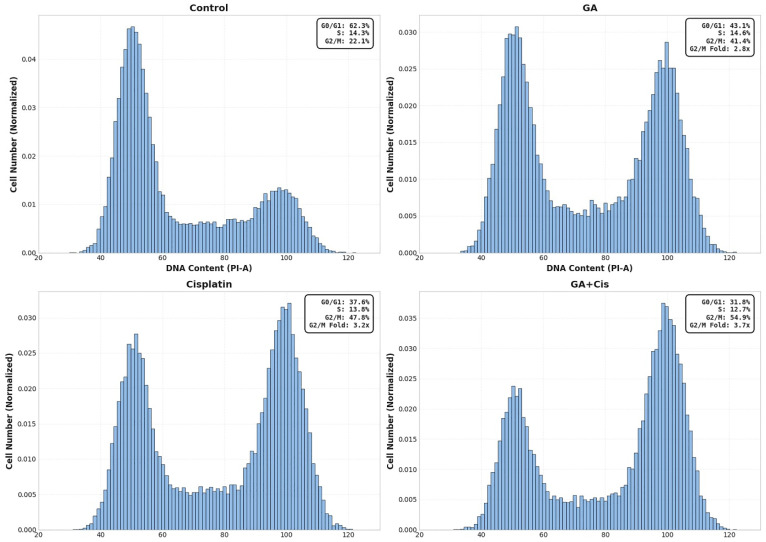
Cell cycle distribution of HeLa cells following treatment with GA (100 µM), Cis (3.5 µM), and their combination (100 µM GA + 3.5 µM Cis) for 48 h. Representative histograms show DNA content following PI staining. Treatment with GA and Cis resulted in an increased proportion of cells in the G2/M phase, with the highest accumulation observed in the combination group. A modest increase in the sub-G1 population was also observed, consistent with apoptotic cell death. Quantification of cell cycle distribution demonstrated progressive enrichment of the G2/M population following single and combination treatments.

Cell cycle distribution analysis demonstrated that treatment with GA (100 µM), Cis (3.5 µM), and their combination (100 µM GA + 3.5 µM Cis) altered the phase distribution of HeLa cells (Figure 5). In the control group, cells were predominantly distributed in the G0/G1 phase (62.3%), with smaller proportions in the S (14.3%) and G2/M (22.1%) phases. Treatment with GA resulted in a decrease in the G0/G1 population (43.1%) and a corresponding increase in the G2/M phase (41.8%). Cis treatment further enhanced this effect, with the G2/M population increasing to 47.8%, accompanied by a reduction in the G0/G1 fraction (37.6%). The most pronounced shift was observed in the combination group, where the G2/M population reached 54.9%, representing approximately a 2.3-fold increase compared to the control group. These findings indicate an accumulation of cells in the G2/M phase following treatment, consistent with altered cell cycle progression under the tested conditions.

### 3.4. NucBlue Immunocytochemistry Staining Findings

**Figure 6:**
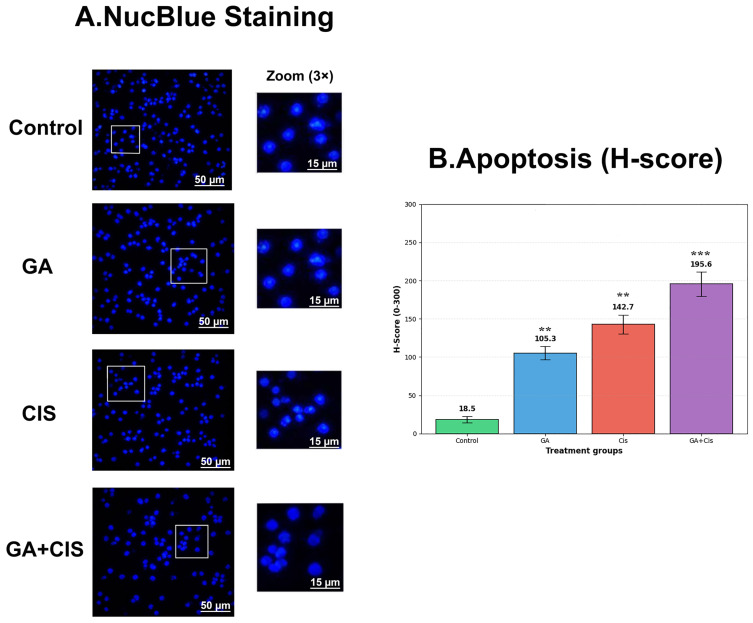
Effects of GA (100 µM), Cis (3.5 µM), and their combination (100 µM GA + 3.5 µM Cis) on nuclear morphology in HeLa cells after 48 h of treatment. (**A**) Representative fluorescence images of NucBlue (Hoechst 33342) staining and boxed regions shown at 3× higher magnification. Control cells exhibited relatively uniform nuclear morphology, whereas treated groups showed nuclear features consistent with apoptotic alterations, including chromatin condensation and increased nuclear shrinkage, which appeared more prominent in the combination treatment group. Scale bars: 50 µm (main images) and 15 µm (zoomed images). (**B**) Semi-quantitative analysis of apoptotic nuclear alterations expressed as H-score (0–300), demonstrating increased apoptosis-associated nuclear changes following single and combination treatments. Data are presented as mean ± SD from three independent biological replicates (*n* = 3). Statistical significance was evaluated relative to the control group (** *p* < 0.01, *** *p* < 0.001).

Nuclear morphology of HeLa cells treated with GA (100 µM), Cis (3.5 µM), and their combination (100 µM GA + 3.5 µM Cis) for 48 h was evaluated using NucBlue (Hoechst 33342) fluorescent staining (Figure 6). Cells in the control group exhibited a homogeneous and uniformly distributed nuclear staining pattern. In contrast, treated groups showed morphological alterations associated with apoptotic changes, including chromatin condensation, nuclear fragmentation, and irregular nuclear morphology. These morphological changes appeared more pronounced in the GA+Cis combination group than in the single-agent treatments. Semi-quantitative analysis performed using ImageJ software indicated a significant increase in the proportion of cells exhibiting apoptotic nuclear features in the combination group relative to the control group (*p* < 0.001).

### 3.5. β-Tubulin Immunocytochemical Staining Findings

**Figure 7:**
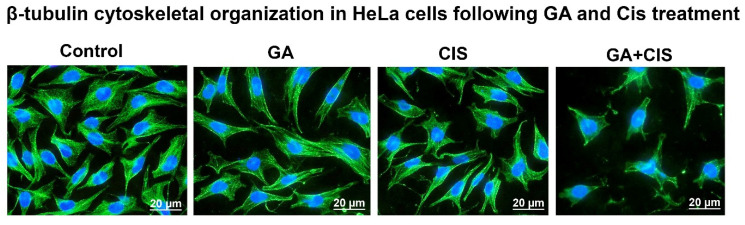
Immunofluorescence staining of β-tubulin (green) and nuclei (Hoechst 33342, blue) in HeLa cells following treatment with GA (100 µM), Cis (3.5 µM), and their combination (100 µM GA + 3.5 µM Cis) for 48 h. Control cells exhibited a well-organized microtubule network, whereas treated groups showed alterations in β-tubulin filament organization, including reduced network continuity and irregular filament distribution. These changes appeared more pronounced in the combination group. Scale bar = 20 µm.

Immunofluorescence staining of β-tubulin (green) combined with nuclear staining (DAPI/Hoechst, blue) was used to evaluate cytoskeletal organization in HeLa cells treated with GA (100 µM), Cis (3.5 µM), and their combination (100 µM GA + 3.5 µM Cis) for 48 h (Figure 7). In the control group, cells exhibited a relatively organized microtubule network with uniform nuclear morphology. In contrast, treatment with GA and Cis was associated with visible alterations in β-tubulin filament organization, including reduced network continuity and irregular filament distribution. These structural changes appeared more pronounced in the combination group, accompanied by nuclear morphological alterations associated with apoptotic features. Semi-quantitative image analysis indicated a significant reduction in β-tubulin staining intensity in the combination group compared to the control group (*p* < 0.01).

### 3.6. Determination of Apoptosis-Related Gene Expression by RT-qPCR

**Figure 8:**
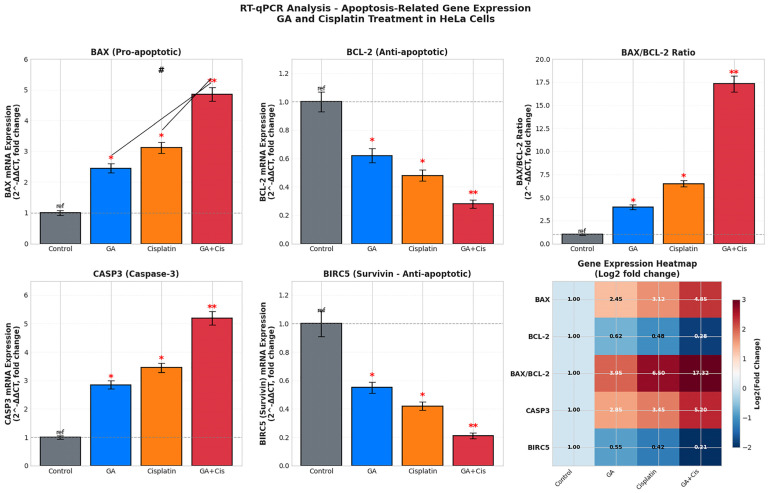
Effects of GA (100 µM), Cis (3.5 µM), and their combination (100 µM GA + 3.5 µM Cis) on the mRNA expression levels of apoptosis-related genes (BAX, BCL-2, CASP3, and BIRC5) in HeLa cells after 48 h of treatment. Gene expression levels were normalized using the 2^−ΔΔCt^ method and are presented as mean ± SD from three independent biological replicates (*n* = 3). Treatment groups showed increased expression of pro-apoptotic genes (BAX and CASP3) and decreased expression of anti-apoptotic genes (BCL-2 and BIRC5) relative to the control group. The BAX/BCL-2 ratio was markedly elevated in the combination group. A heatmap visualization illustrates the overall gene expression patterns across treatment groups. Dashed horizontal lines indicate the normalized control baseline (fold change = 1). Solid lines and the “#” symbol indicate comparisons highlighting the enhanced effect observed in the combination treatment group relative to single-treatment groups. Statistical significance is indicated as * *p* < 0.05, ** *p* < 0.01.

The effects of GA (100 µM), Cis (3.5 µM), and their combination (100 µM GA + 3.5 µM Cis) on apoptosis-related gene expression were evaluated by RT-qPCR after 48 h of treatment (Figure 8). Compared to the control group, both GA and Cis treatments were associated with increased mRNA expression levels of the pro-apoptotic genes BAX and CASP3, along with decreased expression of the anti-apoptotic genes BCL-2 and BIRC5 (*p* < 0.05). The highest BAX expression and the lowest BCL-2 expression were observed in the GA+Cis combination group. Consistently, the BAX/BCL-2 ratio was markedly elevated in the combination group compared to single-agent treatments. CASP3 expression was also increased across all treatment groups, with the greatest elevation observed in the combination group. In contrast, BIRC5 expression showed a progressive decrease following treatment, with the lowest levels detected in the combination group. These expression patterns are compatible with a shift toward pro-apoptotic signaling under the tested conditions. Heatmap visualization further illustrates the coordinated changes in gene expression across treatment groups.

### 3.7. Enzyme Activities

**Figure 9:**
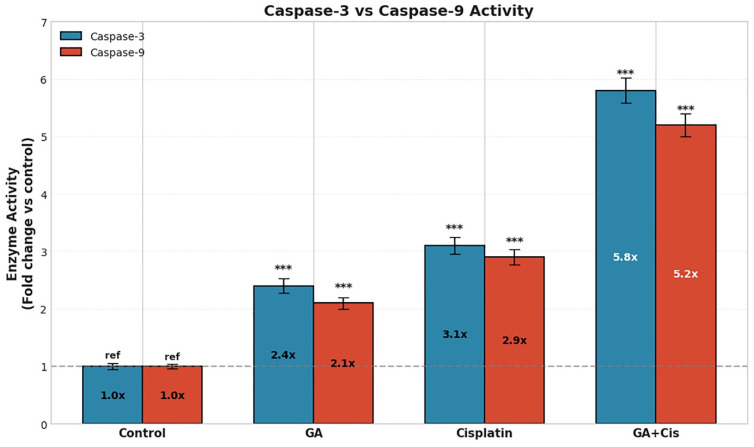
Effects of GA (100 µM), Cis (3.5 µM), and their combination (100 µM GA + 3.5 µM Cis) on caspase-3 and caspase-9 enzyme activities in HeLa cells after 48 h of treatment, measured using a colorimetric assay. Enzyme activities are expressed as fold change relative to the control group. Both single-agent and combination treatments increased caspase-3 and caspase-9 activities compared to control cells, with the highest activation observed in the combination group. The dashed horizontal line indicates the baseline control level (1.0-fold change). Data are presented as mean ± SD from three independent biological replicates (*n* = 3). Statistical significance is indicated as *** *p* < 0.001 vs. control.

Colorimetric activity measurements demonstrated that treatment with GA (100 µM), Cis (3.5 µM), and their combination (100 µM GA + 3.5 µM Cis) for 48 h increased caspase-3 and caspase-9 activities in HeLa cells (Figure 9). Compared to the control group, caspase-3 activity increased 2.4-fold in the GA group, 3.1-fold in the Cis group, and 5.8-fold in the GA+Cis combination group (*p* < 0.001). Similarly, caspase-9 activity increased 2.1-fold in the GA group, 2.9-fold in the Cis group, and 5.2-fold in the combination group (*p* < 0.001). Both caspase-3 and caspase-9 activities were higher in the combination group than in the single-agent treatments. These findings support the involvement of caspase-associated apoptotic processes under the tested treatment conditions.

### 3.8. Cytokine Profile

**Figure 10:**
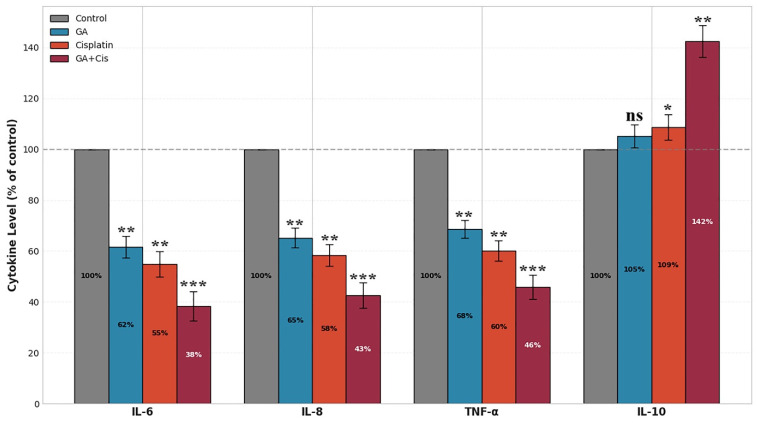
Effects of GA (100 µM), Cis (3.5 µM), and their combination (100 µM GA + 3.5 µM Cis) on cytokine levels (IL-6, IL-8, TNF-α, and IL-10) in HeLa cell culture supernatants after 48 h of treatment. Cytokine levels are expressed as percentages relative to the control group. Treatment with GA and Cis individually reduced the levels of the pro-inflammatory cytokines IL-6, IL-8, and TNF-α, whereas the combination treatment produced a more pronounced reduction. In contrast, IL-10 levels increased following treatment, with the highest elevation observed in the combination group. The dashed horizontal line indicates the baseline control level (100%). Data are presented as mean ± SD from three independent biological replicates (*n* = 3). Statistical significance is indicated as * *p* < 0.05, ** *p* < 0.01, and *** *p* < 0.001 vs. control; ns: not significant.

Cytokine levels in cell culture supernatants were measured using ELISA following treatment with GA (100 µM), Cis (3.5 µM), and their combination (100 µM GA + 3.5 µM Cis) for 48 h (Figure 10). Treatment with GA and Cis was associated with reduced levels of pro-inflammatory cytokines, including IL-6, IL-8, and TNF-α, compared to the control group. Specifically, IL-6 levels decreased by 38.4 ± 4.2% in the GA group, 45.1 ± 5.0% in the Cis group, and 61.7 ± 5.8% in the combination group (*p* < 0.05). Similar reductions were observed for IL-8 and TNF-α, with the greatest decrease detected in the combination group. In contrast, levels of the anti-inflammatory cytokine IL-10 remained relatively unchanged following GA and Cis treatments alone, whereas a significant increase was observed in the combination group (*p* < 0.05). These findings suggest that GA and Cis treatment may be associated with modulation of cytokine profiles under the tested conditions. Cytokine changes were interpreted in the context of cell viability data to minimize potential bias related to treatment-induced differences in cell number.

### 3.9. Potential Targets of GA and Overlapping Genes with Cervical Cancer

**Figure 11:**
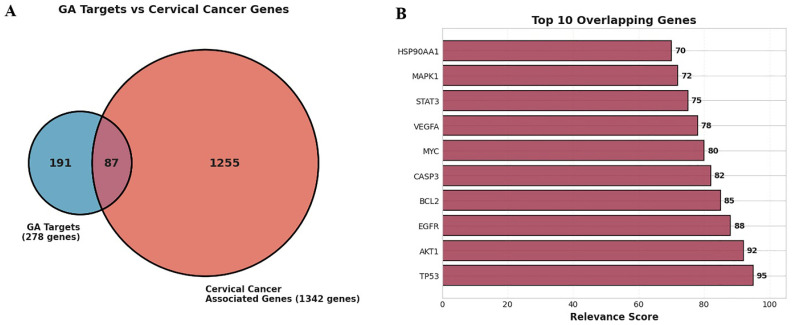
(**A**) Venn diagram showing the overlap between predicted GA targets and cervical cancer-associated genes. A total of 87 overlapping genes were identified. (**B**) Top 10 overlapping genes ranked based on relevance scores obtained from database analysis.

A total of 278 potential targets of GA were predicted using the SwissTargetPrediction platform. In parallel, 1342 genes associated with cervical cancer were compiled from the GeneCards and OMIM databases. Venn diagram analysis identified 87 overlapping genes between predicted GA targets and cervical cancer-associated genes (Figure 11A). Among these overlapping genes, TP53, AKT1, EGFR, BCL2, CASP3, MYC, VEGFA, STAT3, MAPK1, and HSP90AA1 were identified as highly represented genes based on database-derived relevance scores (Figure 11B).

### 3.10. PPI Network and Hub Gene Analysis

**Figure 12:**
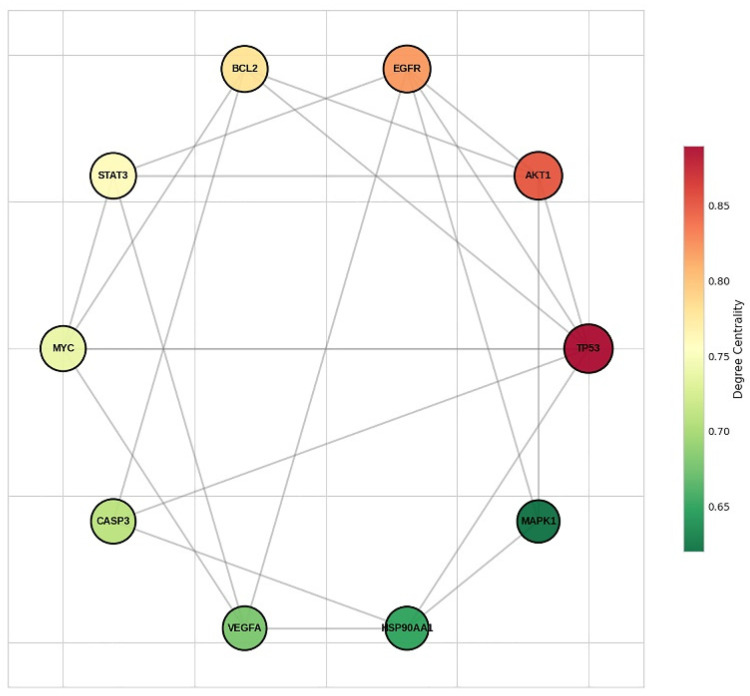
PPI network of the 87 overlapping genes between predicted GA targets and cervical cancer-associated genes. The network was constructed using the STRING database and visualized in Cytoscape. Nodes represent proteins and edges represent protein–protein interactions. Node color indicates degree centrality, with darker colors representing more highly connected (hub) genes.

The PPI network constructed using the STRING database consisted of 87 nodes and 412 edges (Figure 12). Topological analysis performed using the NetworkAnalyzer tool identified the top 10 hub genes based on degree centrality, including TP53, AKT1, EGFR, BCL2, STAT3, MYC, CASP3, VEGFA, HSP90AA1, and MAPK1. Among these, TP53 showed the highest centrality value, suggesting a potentially prominent position within the network topology. Overall, the PPI analysis highlights a set of highly connected genes that may be relevant to the biological effects of GA in cervical cancer.

### 3.11. GO and KEGG Pathway Enrichment Analysis

**Figure 13:**
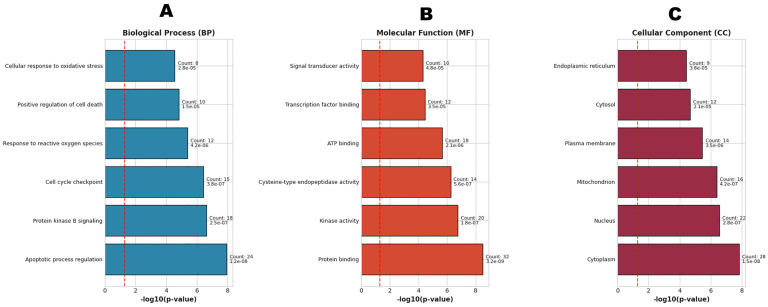
GO enrichment analysis of overlapping genes between predicted GA targets and cervical cancer-associated genes. (**A**) BP, (**B**) MF, and (**C**) CC categories showing significantly enriched GO terms based on adjusted *p*-values.

**Figure 14:**
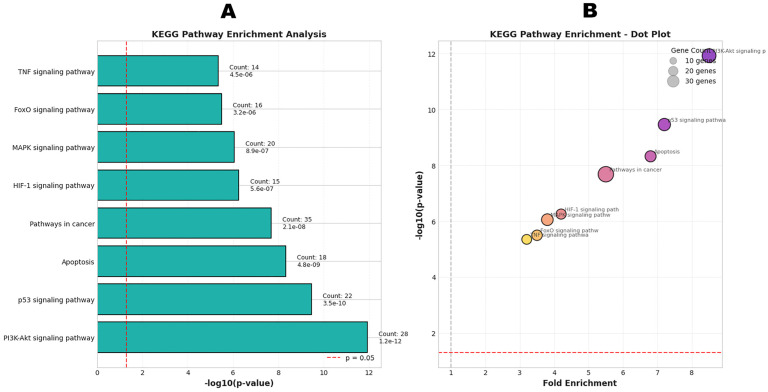
KEGG pathway enrichment analysis of overlapping genes between predicted GA targets and cervical cancer-associated genes. (**A**) Bar plot showing significantly enriched KEGG pathways based on adjusted *p*-values. (**B**) Dot plot illustrating the enrichment of pathways according to fold enrichment and statistical significance.

GO enrichment analysis revealed that the overlapping gene set was significantly associated with multiple biological processes, including regulation of apoptotic processes, protein kinase B signaling, cell cycle checkpoint, and cellular responses associated with oxidative stress (Figure 13). In the MF category, enriched terms included protein binding, kinase activity, and cysteine-type endopeptidase activity, while CC analysis indicated enrichment in compartments such as the nucleus, cytoplasm, and mitochondrion. KEGG pathway enrichment analysis identified several significantly enriched pathways (adjusted *p*-value < 0.05), including the PI3K-Akt signaling pathway, p53 signaling pathway, apoptosis, pathways in cancer, and HIF-1 signaling pathway (Figure 14). Overall, enrichment analysis indicated that the predicted target genes of GA may be associated with multiple cancer-related signaling pathways and biological processes.

## 4. Discussion

This study provides a comprehensive evaluation of the cytotoxic, apoptotic, and molecular effects of GA in combination with Cis in HeLa cervical cancer cells. The findings indicate that the combination treatment is associated with enhanced cytotoxicity compared to single-agent applications, as reflected by reduced IC_50_ values and CI values ranging from 0.61 to 0.92, suggesting synergistic interactions across a range of effect levels. In addition, SI values exceeding 2 at 48 h indicate preferential cytotoxicity toward cancer cells (HeLa) compared to non-tumorigenic HaCaT cells, consistent with previous reports [19,20]. Functionally, the combination treatment was associated with increased apoptotic cell populations, elevated caspase activity, and characteristic nuclear morphological changes. At the molecular level, gene expression analyses revealed a coordinated shift toward pro-apoptotic signaling, including increased BAX and CASP3 expression and decreased BCL2 and BIRC5 levels, along with an elevated BAX/BCL2 ratio. These findings were further supported by cell cycle alterations, including accumulation in the G2/M phase, and by modulation of cytokine profiles under the tested conditions. Bioinformatic analyses additionally suggested that predicted GA target genes may be enriched in multiple cancer-related pathways, including apoptosis and PI3K–Akt signaling. Collectively, these results suggest that the GA+Cis combination may exert multi-faceted biological effects in cervical cancer cells through interconnected cellular and molecular processes. Importantly, the convergence of phenotypic, enzymatic, transcriptional, and computational findings is compatible with a multi-layered biological response rather than a single dominant mechanism.

Notably, several of the signaling pathways identified in the present bioinformatic analyses are also closely associated with the established anticancer mechanisms of Cis. Previous studies have shown that Cis response and Cis resistance are strongly linked to the PI3K/AKT signaling pathway, which regulates cell proliferation, migration, apoptosis, and drug resistance processes in multiple tumor types, including cervical cancer [21,22]. In parallel, GA has been reported to suppress cancer progression through modulation of apoptosis-related and PI3K/Akt-associated signaling pathways, and co-administration of GA with chemotherapeutic agents has been associated with enhanced anticancer efficacy [23]. Consistent with this interpretation, Liang et al. demonstrated that GA potentiated the anticancer activity of Cis in ovarian cancer cells through regulation of the PI3K/AKT/mTOR pathway [24]. Similarly, Mamat et al. reported that the combination of GA and Cis increased apoptotic cell populations in HeLa cells (12.83 ± 1.44% early apoptosis vs. 8.8 ± 1.75% for Cis alone), accompanied by increased ROS production and reduced antioxidant enzyme activity, suggesting enhanced apoptotic stress under combination treatment conditions [25]. In the current study, enrichment of apoptosis-, PI3K–Akt-, p53-, TNF-, and MAPK-related pathways, together with altered BAX, BCL2, CASP3, and BIRC5 expression patterns, may collectively suggest that the enhanced activity observed in the GA+Cis group involves coordinated modulation of overlapping apoptotic and cellular stress signaling networks rather than a single dominant mechanism.

Apoptosis induction is a key determinant of the efficacy of anticancer therapies. In the present study, the GA+Cis combination markedly increased total apoptotic cell populations compared to single-agent treatments, accompanied by distinct nuclear morphological changes such as chromatin condensation and fragmentation. These findings are consistent with previous reports demonstrating the pro-apoptotic effects of GA in various cancer cell models [13,26]. Notably, the increased proportion of late apoptotic cells observed in the combination group suggests progression toward advanced stages of apoptosis under combined treatment conditions. In addition, cell cycle analysis revealed a substantial accumulation of cells in the G2/M phase following GA and Cis treatment, particularly in the combination group. G2/M phase accumulation has frequently been associated with cellular stress responses and DNA damage-related conditions, allowing either repair or progression toward apoptosis [27]. Previous studies have shown that GA can induce G2/M phase accumulation in different cancer cell types [12,28], and the current findings extend this observation by demonstrating a more pronounced effect under combination treatment conditions.

At the molecular level, RT-qPCR analyses demonstrated that GA and Cis treatments were associated with increased expression of pro-apoptotic genes (BAX and CASP3) and decreased expression of anti-apoptotic genes (BCL2 and BIRC5). The substantial increase in the BAX/BCL2 ratio observed in the combination group reflects a shift toward a pro-apoptotic balance, which is widely recognized as a key determinant of cell fate [29]. Survivin (BIRC5), a member of the IAP family, plays a critical role in cell survival and proliferation, and its overexpression has been linked to poor prognosis in multiple cancer types [30]. The observed reduction in BIRC5 expression is therefore consistent with decreased anti-apoptotic signaling. Furthermore, the increased activities of caspase-3 and caspase-9 indicate activation of caspase-dependent apoptotic processes. Caspase-9 is typically associated with mitochondrial apoptotic signaling, while caspase-3 functions as an executioner caspase [31]. Previous studies have also reported increased caspase activity following GA treatment [32,33], and the present findings are consistent with these observations. However, these results should be interpreted as supportive of apoptotic signaling rather than definitive evidence of a specific mechanistic pathway.

The cytokine profile analysis revealed that GA and Cis treatments were associated with decreased levels of pro-inflammatory cytokines (IL-6, IL-8, and TNF-α), while IL-10 levels increased in the combination group. Chronic inflammation plays a significant role in cancer development and progression [34], and cytokines such as IL-6 and TNF-α are central mediators of tumor-associated inflammatory responses [35]. The anti-inflammatory properties of GA have been reported previously [36,37], and the present findings suggest that GA may modulate cytokine levels under in vitro conditions. However, cytokine measurements in cell culture supernatants may be influenced by treatment-related changes in cell viability, and therefore these findings should be interpreted with caution.

Bioinformatic analyses provided exploratory insight into the potential molecular context of GA activity. A total of 87 overlapping genes were identified between predicted GA targets and cervical cancer-associated genes, including TP53, AKT1, EGFR, BCL2, CASP3, MYC, VEGFA, STAT3, MAPK1, and HSP90AA1. These genes are known to be involved in key cancer-related processes such as apoptosis, proliferation, angiogenesis, and signal transduction. PPI network analysis indicated that TP53 occupies a central position within the network, consistent with its well-established role in tumor suppression and regulation of apoptosis [38]. Previous studies have also suggested that GA may influence TP53-related signaling [39]. However, these findings should be considered hypothesis-generating and require further experimental validation.

KEGG pathway enrichment analysis revealed that the overlapping gene set was associated with multiple cancer-related pathways, including PI3K–Akt signaling, p53 signaling, apoptosis, pathways in cancer, and HIF-1 signaling pathways. The PI3K–Akt pathway has been widely implicated in regulating cell survival, proliferation, and resistance to apoptosis, and its dysregulation is common in various cancers [40]. GA has been reported to influence this pathway in previous studies [41,42]. Similarly, enrichment of the HIF-1 signaling pathway suggests a potential association with cellular responses to hypoxia. However, enrichment analysis reflects statistical associations rather than direct mechanistic evidence and should be interpreted accordingly.

Despite the comprehensive experimental design, several limitations should be considered when interpreting the findings of this study. First, the experiments were conducted using a single cervical cancer cell line (HeLa), which may limit the generalizability of the results across different tumor subtypes. Second, molecular analyses were primarily based on mRNA expression and enzyme activity measurements without protein-level validation, which may restrict mechanistic interpretation. Third, causal relationships between the observed molecular alterations and apoptotic outcomes were not directly investigated using pathway-specific inhibitors or gene-silencing approaches. In addition, upstream events potentially associated with oxidative stress-related signaling, including ROS generation, mitochondrial dysfunction, and DNA damage responses, were not directly evaluated. Cytokine measurements may have also been influenced by treatment-related differences in cell viability. Furthermore, the bioinformatic analyses were predictive in nature and should therefore be considered hypothesis-generating rather than confirmatory. Finally, the absence of in vivo validation limits the translational relevance of the present findings.

Future studies should focus on validating these findings in multiple cervical cancer models, including additional cell lines and in vivo systems. Protein-level validation of key apoptotic and signaling molecules would strengthen mechanistic interpretation. Functional studies using pharmacological inhibitors or gene-silencing approaches could help clarify causal pathways. Investigating whether GA can reduce Cis-associated toxicity while maintaining therapeutic efficacy would also be of significant clinical interest. Furthermore, integrative omics approaches, such as transcriptomics and proteomics, may provide a more comprehensive understanding of the multi-target effects of GA. Finally, pharmacokinetic and bioavailability studies are required to support the clinical translation of GA-based combination strategies.

## 5. Conclusions

In conclusion, the present study demonstrates that the combination of GA and Cis is associated with enhanced anticancer activity in HeLa cervical cancer cells compared to single-agent treatments. This effect was supported by multiple complementary findings, including reduced cell viability, synergistic interaction indicated by CI values below 1, and increased selectivity toward cancer cells at 48 h. The combination treatment was further associated with increased apoptotic cell populations, elevated caspase-3 and caspase-9 activities, and nuclear morphological changes consistent with apoptosis. At the molecular level, gene expression analysis revealed a shift toward pro-apoptotic signaling, characterized by increased BAX and CASP3 expression, decreased BCL2 and BIRC5 levels, and an elevated BAX/BCL2 ratio. In addition, cell cycle analysis indicated accumulation in the G2/M phase, while cytokine profiling suggested modulation of inflammatory mediators under the tested conditions. Bioinformatic analyses further suggested potential associations with multiple cancer-related pathways, including apoptosis and PI3K–Akt signaling, highlighting the multi-target nature of GA.

Taken together, these findings suggest that GA may enhance the biological activity of Cis in cervical cancer cells through interconnected cellular and molecular processes. However, these results are based on in vitro and predictive analyses, and further studies, including in vivo validation and protein-level investigations, are required to confirm the therapeutic potential and clinical relevance of this combination strategy.

## Data Availability

The original contributions presented in this study are included in the article. Further inquiries can be directed at the corresponding authors.

## Abbreviations

AKT1	AKT Serine/Threonine Kinase 1
ANOVA	Analysis of Variance
ATP	Adenosine Triphosphate
BAX	BCL2-Associated X Protein
BCL2	B-Cell Lymphoma 2
BP	Biological Process
BIRC5	Baculoviral IAP Repeat Containing 5 (Survivin)
CC	Cellular Component
CI	Combination Index
Cis	Cisplatin
DMEM	Dulbecco’s Modified Eagle Medium
DMSO	Dimethyl Sulfoxide
ELISA	Enzyme-Linked Immunosorbent Assay
EGFR	Epidermal Growth Factor Receptor
FBS	Fetal Bovine Serum
GA	Gallic Acid
GO	Gene Ontology
HIF-1	Hypoxia-Inducible Factor 1
HSP90AA1	Heat Shock Protein 90 Alpha Family Class A Member 1
IC_50_	Half-Maximal Inhibitory Concentration
IAP	Inhibitor of Apoptosis Protein
IL	Interleukin
KEGG	Kyoto Encyclopedia of Genes and Genomes
MAPK1	Mitogen-Activated Protein Kinase 1
MF	Molecular Function
MTT	3-(4,5-Dimethylthiazol-2-yl)-2,5-Diphenyltetrazolium Bromide
MYC	MYC Proto-Oncogene
OMIM	Online Mendelian Inheritance in Man
PBS	Phosphate-Buffered Saline
PPI	Protein–Protein Interaction
qPCR	Quantitative Polymerase Chain Reaction
ROS	Reactive Oxygen Species
RT-qPCR	Reverse Transcription Quantitative Polymerase Chain Reaction
SD	Standard Deviation
SI	Selectivity Index
STAT3	Signal Transducer and Activator of Transcription 3
STRING	Search Tool for the Retrieval of Interacting Genes/Proteins
TP53	Tumor Protein p53
TNF-α	Tumor Necrosis Factor Alpha
VEGFA	Vascular Endothelial Growth Factor A
